# Risk factors analysis and validation of early treatment non-response in children with primary ITP: a multicenter retrospective cohort study

**DOI:** 10.3389/fimmu.2026.1983623

**Published:** 2026-10-07

**Authors:** Mingwei Li, Mei Yan

**Affiliations:** Department of Pediatric Center, The First Affiliated Hospital of Xinjiang Medical University, Urumqi, Xinjiang Uyghur Autonomous Region, China

**Keywords:** ana, children, PLR, prediction model, primary immune thrombocytopenia

## Abstract

**Objective:**

To identify factors associated with early non-response to first-line treatment in children with primary immune thrombocytopenia (ITP), and to develop and externally validate a prediction model.

**Methods:**

This multicenter retrospective cohort study included 335 children with newly diagnosed primary ITP as the derivation cohort, including 255 responders and 80 non-responders. Clinical data obtained after admission were collected. Candidate variables were screened using correlation analysis and least absolute shrinkage and selection operator (LASSO) regression, followed by multivariable logistic regression to identify factors associated with early treatment non-response. Model discrimination was evaluated using the area under the receiver operating characteristic curve (AUC). An independent external validation cohort of 78 children was additionally included to assess model discrimination.

**Results:**

Among the 335 children, 255 (76.1%) achieved a treatment response within 14 days after first-line therapy, whereas 80 (23.9%) did not. Multivariable analysis showed that older age (OR = 1.769, 95% CI: 1.629–1.920, P < 0.001), higher platelet-to-lymphocyte ratio (PLR) (OR = 1.622, 95% CI: 1.112–2.367, P = 0.012), and antinuclear antibody (ANA) positivity (OR = 3.142, 95% CI: 1.626–6.071, P < 0.001) were independently associated with early treatment non-response. The combined model incorporating age, PLR, and ANA achieved an AUC of 0.831 (95% CI: 0.778–0.885) in the derivation cohort and 0.718 (95% CI: 0.591–0.845) in the independent external validation cohort. Calibration was acceptable in both cohorts, with Hosmer–Lemeshow P values of 0.609 and 0.220 and Brier scores of 0.129 and 0.137, respectively.

**Conclusion:**

Older age, higher PLR, and ANA positivity were independently associated with early non-response to first-line treatment within 14 days in children with newly diagnosed primary ITP. These findings may help identify children at high risk of treatment non-response and support individualized monitoring and treatment strategies.

## 1 Introduction

Primary immune thrombocytopenia (ITP) is one of the most common acquired immune-mediated bleeding disorders in children. Its pathogenesis involves multiple mechanisms, including autoantibody-mediated platelet destruction, impaired platelet production, and T-cell immune dysregulation (1, 2). The annual incidence of acute ITP in children is approximately 1.9–6.4 per 100, 000, although reported incidence rates vary across countries and regions (3, 4). Previous studies have reported incidence rates of approximately 4.2–8.8 per 100, 000 children in countries and regions including China and the United States (5–7). Although childhood ITP is self-limiting in most cases and approximately 80% of patients achieve remission during the disease course, about 20%–25% progress to persistent or chronic ITP (8, 9). Bleeding is the major clinical risk of ITP, ranging from petechiae and purpura to mucosal bleeding, while a small proportion of patients may develop life-threatening intracranial hemorrhage (10). The incidence of intracranial hemorrhage in pediatric ITP is approximately 0.1%–0.4%, and most related clinical events occur during the early stage of the disease (11). Therefore, early risk identification and assessment of treatment response at the time of initial diagnosis are important for the clinical management of pediatric ITP.

Currently, glucocorticoids and intravenous immunoglobulin (IVIG) remain the most commonly used first-line treatments for children with ITP (12). Although most patients achieve remission, some require further or second-line therapy, which may increase the risks of bleeding, drug exposure, and healthcare burden (13). Previous studies have suggested that age, bleeding manifestations, platelet-related parameters, T-lymphocyte subsets, complement levels, and autoantibodies may be associated with treatment response or disease persistence in pediatric ITP (14, 15). In particular, ANA and other autoantibody abnormalities have been associated with the immune phenotype and short-term treatment response of childhood ITP (16). In recent years, hematological inflammatory indices such as the neutrophil-to-lymphocyte ratio (NLR) and platelet-to-lymphocyte ratio (PLR) have also increasingly been used to evaluate the immune-inflammatory status of ITP (17). However, reliable clinical indicators for predicting first-line treatment response in pediatric ITP remain limited, and dependable pretreatment predictors of response to different first-line therapies are still lacking (18). Furthermore, existing studies have mainly focused on individual treatment modalities or long-term progression to chronic ITP, and independent external validation remains relatively limited.

Therefore, this multicenter retrospective study included a cohort of children with primary ITP and evaluated clinical characteristics, hematological parameters, immunological markers, and inflammation-related indices to identify factors associated with early treatment response. We further assessed the predictive value of these factors in an independent external validation cohort. The findings of this study are intended to provide evidence for clinical risk assessment and individualized treatment and follow-up strategies.

## 2 Materials and methods

### 2.1 Study population: enrollment and follow-up

This study was conducted as a multicenter, retrospective, observational cohort study involving children with newly diagnosed primary immune thrombocytopenia (ITP). The study population was recruited from the First Affiliated Hospital of Xinjiang Medical University and Kashi First People’s Hospital. Children treated at the First Affiliated Hospital of Xinjiang Medical University between January 2023 and June 2025 were included in the derivation cohort for candidate predictor selection and prediction model development. Subsequently, an independent cohort from Kashi First People’s Hospital during the same period was used as the external validation cohort to evaluate the predictive performance of the final model. The inclusion criteria were as follows: (1) patients who met the diagnostic criteria for immune thrombocytopenia; (2) age at disease onset <16 years; (3) no previous treatment related to ITP before enrollment; (4) complete clinical records with available baseline clinical and laboratory data required for the study; and (5) informed consent obtained from the patients’ guardians, with approval from the Medical Ethics Committee of the First Affiliated Hospital of Xinjiang Medical University. The exclusion criteria were as follows: (1) patients diagnosed with secondary thrombocytopenia; (2) patients with malignant tumors; (3) patients with hereditary thrombocytopenia; (4) patients who discontinued treatment for unknown reasons or had incomplete hospitalization records. The external validation cohort applied the same inclusion criteria, exclusion criteria, and variable definitions as the derivation cohort, with the same enrollment period from January 2023 to June 2025.

The index time point was defined as the time of initial diagnosis of primary ITP before initiation of any related treatment. Baseline demographic characteristics, clinical manifestations, hematological parameters, immunological indicators, and inflammatory laboratory parameters were collected before treatment initiation. All patients were followed for 14 days after first-line treatment, and outcomes were determined according to the predefined treatment response criteria.

### 2.2 Definitions

The diagnosis of primary immune thrombocytopenia (ITP) was established according to the diagnostic criteria specified in the Diagnosis and Treatment Specifications for Primary Immune Thrombocytopenia in Children (2019 Edition) (19) and the updated 2021 adapted guideline for pediatric ITP (20). ITP is an acquired immune-mediated disorder characterized mainly by decreased peripheral blood platelet counts after excluding other identifiable causes of thrombocytopenia. The primary outcome of this study was early treatment response within 14 days after initiation of first-line treatment in children with newly diagnosed primary ITP. The 14-day time point was selected based on its common use in pediatric ITP treatment studies as an early assessment point to identify non-responders and allow timely treatment modification (20). Treatment response was evaluated according to platelet count levels and clinical bleeding manifestations after treatment and was classified into complete response (CR), response (R), and non-response (NR). Complete response (CR) was defined as a platelet count ≥100 × 10^9^/L after treatment with no bleeding manifestations. Response (R) was defined as a platelet count ≥30 × 10^9^/L after treatment, with at least a twofold increase from the baseline platelet count and absence of bleeding manifestations. Patients who met either the CR or R criteria were categorized into the responder group (R group), whereas patients who failed to meet either criterion were categorized into the non-responder group (NR group). Therefore, the primary outcome of this study was whether children with newly diagnosed primary ITP achieved an early treatment response within 14 days after initiation of first-line treatment.

Dry bleeding was defined as bleeding involving the skin or mucosa without clinically significant active hemorrhage. According to clinical records, manifestations included petechiae, purpura, or ecchymosis. Wet bleeding was defined as clinically evident mucosal bleeding, including epistaxis, gingival bleeding, gastrointestinal bleeding, genitourinary bleeding, or other mucosal hemorrhagic manifestations documented at initial presentation. Antinuclear antibody (ANA) was detected using the indirect immunofluorescence assay (IIFA) based on HEp-2 cell substrates. ANA results were reported as antibody titers, and an ANA titer ≥1:320 was defined as ANA positivity. The cutoff of ≥1:320 was selected based on previous pediatric ITP studies, in which a median ANA titer of 1:320 has been reported, and high-titer ANA positivity has been associated with immune dysregulation and disease progression (21, 22). The 2021 Chinese guideline for pediatric ITP recommends autoimmune serological testing for persistent/chronic ITP but does not specify a fixed ANA threshold (20). ANA status was analyzed as a categorical variable in subsequent statistical analyses. The neutrophil-to-lymphocyte ratio (NLR) was calculated as the absolute neutrophil count divided by the absolute lymphocyte count. The platelet-to-lymphocyte ratio (PLR) was calculated as the platelet count divided by the absolute lymphocyte count. The monocyte-to-lymphocyte ratio (MLR) was calculated as the absolute monocyte count divided by the absolute lymphocyte count. The systemic immune-inflammation index (SII) was calculated using platelet, neutrophil, and lymphocyte counts, whereas the systemic inflammation response index (SIRI) was calculated based on neutrophil, monocyte, and lymphocyte counts.

First-line treatment regimens were recorded, including corticosteroids (prednisone 2 mg/kg/day for 4–7 days, followed by tapering) or intravenous immunoglobulin (IVIG, 1 g/kg/day for 2 days), consistent with the 2019 Chinese specifications and the 2021 adapted guideline for pediatric ITP (19, 20). Both centers followed the same standardized first-line treatment protocol.

### 2.3 Data collection

Baseline demographic characteristics, clinical characteristics, hematological parameters, immunological indicators, and inflammatory-related parameters were retrospectively collected from the electronic medical record systems of the participating centers. All candidate predictors were obtained from measurements performed at the initial diagnosis of ITP and before initiation of first-line treatment. Demographic and clinical characteristics included age, sex, ethnicity, residence, season of disease onset, recent vaccination history, maternal pregnancy-related factors, family history of ITP, dry bleeding, wet bleeding, and other clinical characteristics. Ethnicity was categorized as Uyghur, Han, or other ethnic groups. Season of disease onset was categorized as spring, summer, autumn, or winter. Routine hematological parameters included white blood cell count (WBC), neutrophil count (Neu), lymphocyte count (Lym), monocyte count (Mon), eosinophil count (Eos), basophil count (Bos), red blood cell count (RBC), hemoglobin (Hb), red cell distribution width (RDW), and platelet count (PLT). Biochemical, immunological, and inflammatory-related parameters included folic acid, vitamin B12, serum iron, erythropoietin (EPO), CD4^+^ T-cell percentage, CD8^+^ T-cell percentage, CD4/CD8 ratio, complement components C3 and C4, C-reactive protein (CRP), interleukin-6 (IL-6), vitamin D, and antinuclear antibodies (ANA). In addition, inflammation-related derived indices, including the neutrophil-to-lymphocyte ratio (NLR), platelet-to-lymphocyte ratio (PLR), monocyte-to-lymphocyte ratio (MLR), systemic immune-inflammation index (SII), and systemic inflammation response index (SIRI), were calculated. All collected variables were obtained from the initial examinations performed after admission.

### 2.4 Statistical methods

Statistical analyses were performed using R software (version 4.6.1). This study was reported following the TRIPOD (Transparent Reporting of a multivariable prediction model for Individual Prognosis Or Diagnosis) statement. Sample size was assessed using the framework proposed by Riley et al (23, 24). Based on the observed event rate (23.9%) and 3 predictor parameters, the study meets the recommended events-per-variable (EPV) threshold (EPV > 10) and the minimum sample size criteria for prediction model development. Missing data were examined for all candidate variables. Multiple imputation by chained equations (MICE) was performed using the ‘mice’ package with 5 imputed datasets. Results were pooled using Rubin’s rules. No patients were excluded due to missing data. The distribution of continuous variables was assessed using the Shapiro–Wilk test. Continuous variables with non-normal distributions were presented as medians and interquartile ranges, expressed as median (Q1, Q3), whereas categorical variables were presented as frequencies and percentages [n (%)]. Baseline characteristics were compared between the responder group and the non-responder group. Continuous variables were compared using the Wilcoxon rank-sum test. Categorical variables were analyzed using Pearson’s chi-squared test or Fisher’s exact test, as appropriate. Predictor selection was performed using a combination of correlation analysis and least absolute shrinkage and selection operator (LASSO) regression to reduce the impact of multicollinearity among variables and identify clinically relevant predictors. Variables selected through the above procedures were subsequently entered into a multivariable logistic regression model. The discrimination ability of the model was evaluated using the area under the receiver operating characteristic curve (AUC). External validation mainly focused on evaluating the discrimination ability of the model using AUC. Model calibration was assessed using calibration curves, the Hosmer–Lemeshow goodness-of-fit test, and the Brier score. Decision curve analysis (DCA) was used to assess clinical utility across a range of threshold probabilities.

## 3 Results

### 3.1 Baseline characteristics

This retrospective cohort study included 335 pediatric patients, of whom 255 were classified into the R group and 80 (23.88%) into the NR group (**Figure 1**). There were no significant differences in sex or BMI between the two groups (P > 0.05). The NR group was older than the R group, with median ages of 6.60 (4.00–9.50) and 4.00 (1.50–7.00) years, respectively (P < 0.001). In addition, the PLR was higher in the NR group than in the R group [1.11 (0.82–1.78) vs. 0.84 (0.45–1.44), P < 0.001] (**Table 1**). Significant between-group differences were also observed in several clinical and laboratory variables, including city residence, house renovation history, wet bleeding, neutrophil count (Neu), PCTp, C3, IgA, vitamin D (Vit_D), SIRI, and ANA (all P < 0.05). Further analysis showed that folic acid and Fe2 levels also differed significantly between the two groups (P < 0.05) (**Supplementary Table 1**).

**Figure 1:**
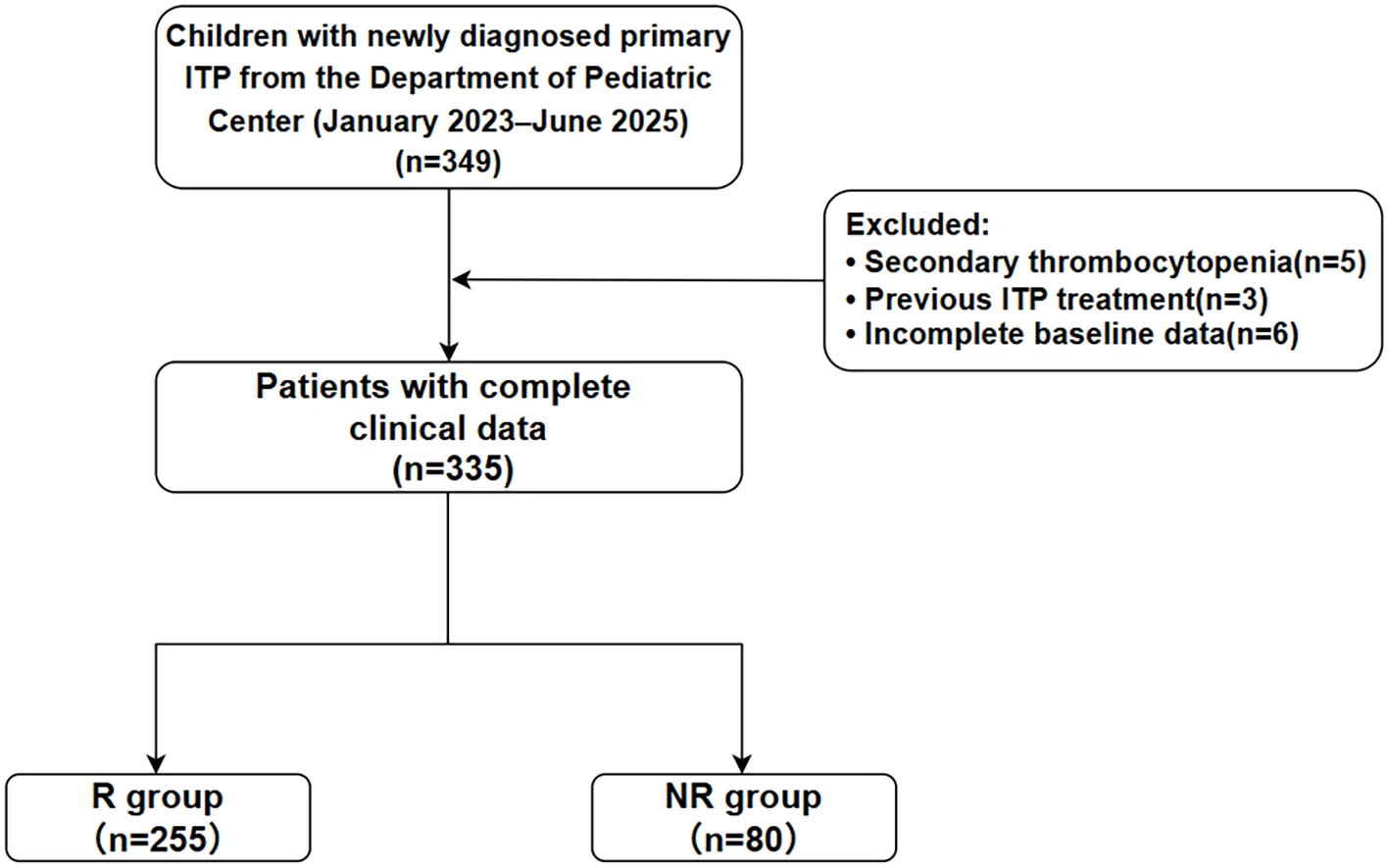
Patient enrollment and grouping.

**Table 1:** Comparison of baseline characteristics between the R group and NR group.

Characteristic	Overall N = 335	R group N = 255	NR group N = 80	p-value
Sex				0.719
Male	191 (57.0%)	144 (56.5%)	47 (58.8%)	
Female	144 (43.0%)	111 (43.5%)	33 (41.3%)	
Age	4.50 (2.00, 7.50)	4.00 (1.50, 7.00)	6.60 (4.00, 9.50)	<0.001
BMI	16.10 (14.90, 17.90)	16.00 (14.80, 17.70)	16.25 (15.00, 18.50)	0.461
City				0.003
No	241 (71.9%)	194 (76.1%)	47 (58.8%)	
Yes	94 (28.1%)	61 (23.9%)	33 (41.3%)	
House_renovation				0.038
NO	301 (89.9%)	234 (91.8%)	67 (83.8%)	
YES	34 (10.1%)	21 (8.2%)	13 (16.3%)	
Dry_bleeding				0.034
No	70 (20.9%)	60 (23.5%)	10 (12.5%)	
Yes	265 (79.1%)	195 (76.5%)	70 (87.5%)	
Wet_bleeding				<0.001
No	215 (64.2%)	176 (69.0%)	39 (48.8%)	
Yes	120 (35.8%)	79 (31.0%)	41 (51.3%)	
WBC	8.12 (6.20, 10.10)	8.07 (6.15, 9.86)	8.53 (6.25, 10.77)	0.340
Neu	3.25 (1.94, 4.66)	3.01 (1.86, 4.47)	3.60 (2.65, 5.09)	0.003
RBC	4.51 (4.11, 4.80)	4.49 (4.11, 4.75)	4.58 (4.18, 4.86)	0.160
Hb	120.00 (107.00, 130.00)	119.00 (107.00, 129.00)	123.50 (109.00, 132.50)	0.104
RDW	13.20 (12.50, 14.10)	13.10 (12.40, 14.10)	13.20 (12.60, 14.20)	0.529
PLT	22.00 (10.00, 42.00)	24.00 (11.00, 44.00)	19.50 (7.50, 35.00)	0.077
MPV	10.80 (9.10, 12.30)	10.63 (9.20, 12.20)	11.20 (8.85, 12.80)	0.539
PCTp	0.02 (0.01, 0.05)	0.03 (0.01, 0.06)	0.02 (0.00, 0.04)	0.038
PDW	16.50 (13.10, 17.90)	16.60 (13.30, 17.90)	15.72 (11.45, 18.05)	0.200
CD4	35.70 (30.10, 41.20)	35.20 (29.40, 41.80)	35.95 (32.10, 41.00)	0.641
CD8	26.60 (21.90, 31.90)	26.40 (21.60, 32.00)	27.35 (22.30, 31.35)	0.718
CD4/CD8	1.30 (1.00, 1.80)	1.30 (1.00, 1.80)	1.35 (1.00, 1.70)	0.945
C3	0.81 (0.69, 0.93)	0.80 (0.68, 0.92)	0.85 (0.75, 0.95)	0.004
C4	0.16 (0.13, 0.21)	0.16 (0.13, 0.21)	0.16 (0.13, 0.19)	0.607
IgG	19.70 (10.50, 28.10)	19.50 (9.72, 27.60)	19.80 (11.90, 28.40)	0.268
IgA	0.85 (0.49, 1.35)	0.80 (0.46, 1.27)	1.10 (0.64, 1.42)	0.007
CRP	5.00 (5.00, 8.80)	5.00 (5.00, 8.80)	5.00 (5.00, 7.85)	0.698
IL_6	3.23 (1.50, 6.34)	3.43 (1.50, 7.05)	2.72 (1.50, 4.60)	0.124
Vit_D	55.30 (36.78, 78.26)	57.50 (38.21, 79.36)	47.20 (33.54, 65.65)	0.013
PLR	0.94 (0.50, 1.49)	0.84 (0.45, 1.44)	1.11 (0.82, 1.78)	<0.001
NLR	6.46 (2.65, 14.08)	6.60 (2.66, 14.95)	5.84 (1.88, 12.63)	0.402
MLR	0.17 (0.13, 0.24)	0.16 (0.12, 0.24)	0.18 (0.13, 0.22)	0.334
SII	18.74 (7.29, 45.84)	17.41 (6.72, 47.34)	23.70 (7.98, 42.04)	0.414
SIRI	0.53 (0.27, 0.92)	0.49 (0.23, 0.88)	0.57 (0.38, 1.07)	0.016
TGAb	236.40 (18.25, 466.10)	236.40 (16.82, 445.60)	237.70 (21.39, 545.65)	0.166
TPOAb	78.62 (14.55, 120.20)	76.40 (14.16, 121.70)	82.47 (17.66, 115.90)	0.960
ANA				0.019
No	125 (37.3%)	104 (40.8%)	21 (26.3%)	
Yes	210 (62.7%)	151 (59.2%)	59 (73.8%)	

WBC, white blood cell count; NEU, neutrophil count; RBC, red blood cell count; HB, hemoglobin; RDW, red cell distribution width; PLT, platelet count; MPV, mean platelet volume; PCT, plateletcrit; PDW, platelet distribution width; CD4, CD4^+^ T-cell percentage; CD8, CD8^+^ T-cell percentage; C3, complement component 3; C4, complement component 4; IgG, immunoglobulin G; IgA, immunoglobulin A; CRP, C-reactive protein; IL-6, interleukin-6; Vit_D, vitamin D; PLR, platelet-to-lymphocyte ratio; NLR, neutrophil-to-lymphocyte ratio; MLR, monocyte-to-lymphocyte ratio; SII, systemic immune-inflammation index; SIRI, systemic inflammation response index; TGAb, thyroglobulin antibody; TPOAb, thyroid peroxidase antibody; ANA, antinuclear antibody.

### 3.2 Collinearity analysis

Based on previous studies and the existing evidence, 22 potential risk factors were prespecified for subsequent model development. Of these, 18 continuous variables were assessed for pairwise associations using Spearman rank correlation coefficients because several laboratory variables showed markedly skewed distributions, whereas four categorical variables (sex, dry bleeding, wet bleeding, and ANA) were not included in the correlation analysis. Several strong correlations were identified among the continuous variables, defined as an absolute correlation coefficient of ≥0.70 (**Figure 2**). To reduce redundant information and minimize potential multicollinearity before subsequent model fitting, four highly correlated variables, namely PLT, NLR, PCT, and SII, were excluded based on the correlation structure and clinical relevance. The remaining 18 candidate variables were subsequently entered into the least absolute shrinkage and selection operator (LASSO) regression analysis for further variable selection.

**Figure 2:**
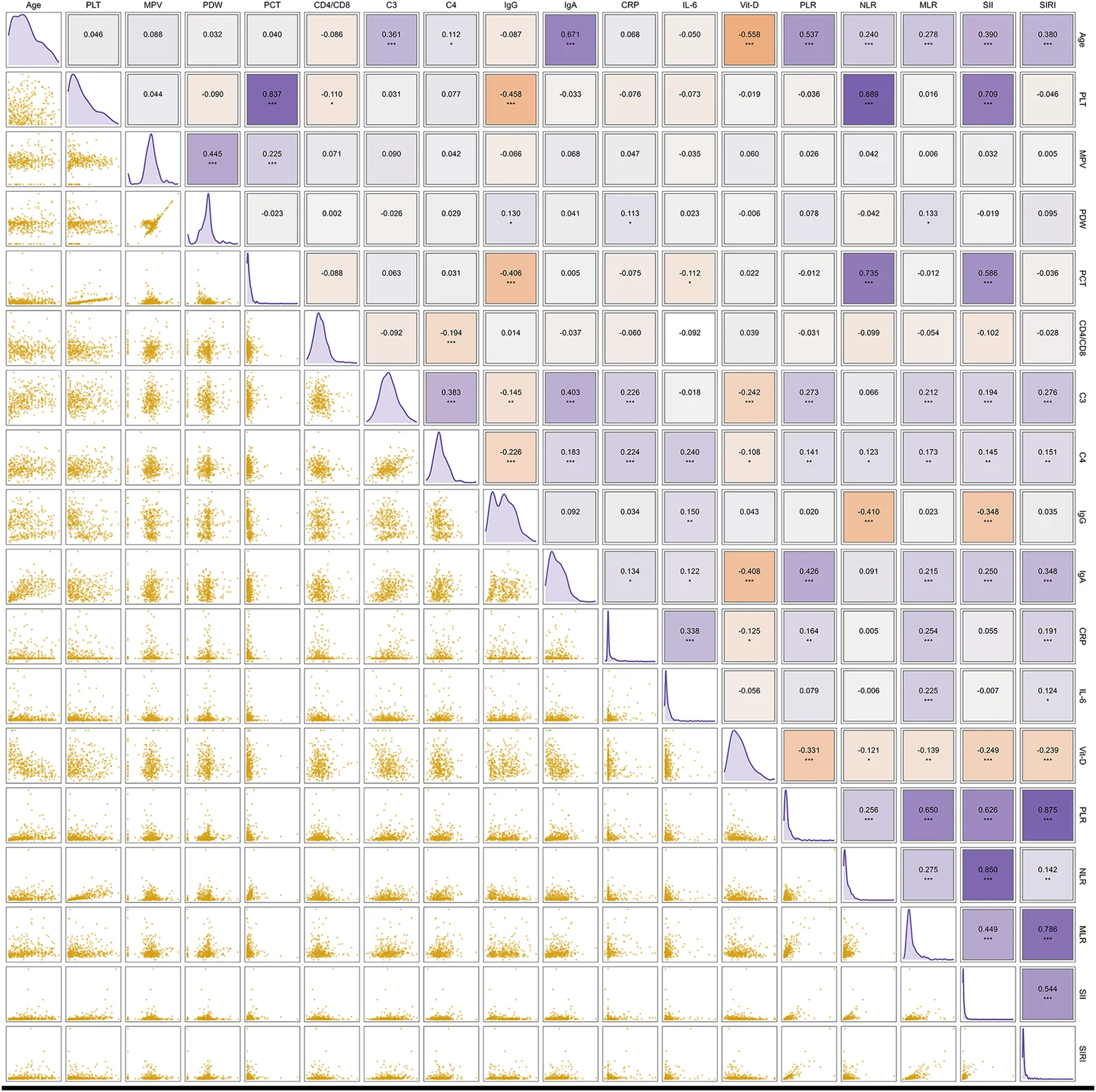
Pairwise Spearman correlation matrix of continuous variables. Diagonal panels show variable distributions; lower panels show scatter plots with fitted trend lines; upper panels show Pearson correlation coefficients. Asterisks indicate statistical significance: P < 0.05 (*), P < 0.01 (**), and P < 0.001 (***).

### 3.3 LASSO regression

After collinearity assessment, the 18 retained candidate variables were further entered into the least absolute shrinkage and selection operator (LASSO) regression analysis. Ten-fold cross-validation was used to determine the optimal penalty parameter. The minimum binomial deviance corresponded to λ_min = 0.022, whereas the value determined according to the one-standard-error (1-SE) rule was λ_1se = 0.057 (**Supplementary Figure 1**). At λ_1se, four variables had non-zero regression coefficients, including Age, PDW, PLR, and ANA. These four variables were therefore retained for subsequent multivariable logistic regression analysis.

### 3.4 Multivariable logistic regression analysis

Following LASSO regression analysis, four predictors—Age, PLR, PDW, and ANA —were entered into a multivariable logistic regression analysis. The results showed that Age, PLR, and ANA were associated with an increased risk of treatment non-response, whereas PDW was not significantly associated with treatment non-response and was therefore excluded from the final prediction model (**Supplementary Table S2**). The final multivariable logistic regression model was subsequently refitted using Age, PLR, and ANA. The results showed that older age was independently associated with an increased risk of treatment non-response (OR = 1.769, 95% CI: 1.629–1.920, P < 0.001). Similarly, higher PLR was associated with an increased risk of treatment non-response (OR = 1.622, 95% CI: 1.112–2.367, P = 0.012), and ANA positivity was independently associated with an increased risk of treatment non-response (OR = 3.142, 95% CI: 1.626–6.071, P < 0.001) (**Table 2**).

**Table 2:** Multivariable logistic regression analysis of early treatment non-response in the R and NR groups.

Variable	Beta	SE	OR	95%CI	P
(Intercept)	-3.499	0.416	0.030	0.013-0.068	<0.001
PLR	0.484	0.193	1.622	1.112-2.367	0.012
Age	0.570	0.042	1.769	1.629-1.920	<0.001
ANA	1.145	0.336	3.142	1.626-6.071	<0.001

### 3.5 Model performance and validation

Receiver operating characteristic (ROC) curve analysis was performed to evaluate the discriminative performance of the individual predictors and the combined model. In the development cohort, the AUC for Age, ANA, and PLR were 0.665, 0.777, and 0.626, respectively. The combined model incorporating the selected predictors demonstrated improved discriminative ability, with an AUC of 0.831 (**Figure 3**). These findings indicated that the combined model provided better discrimination than any individual predictor alone. The final model was subsequently externally validated in an independent cohort from Kashi First People’s Hospital. The results showed that the model maintained good discriminative ability, with an AUC of 0.718 (95% CI: 0.591–0.845), compared with an AUC of 0.831 (95% CI: 0.778–0.885) in the development cohort (**Figure 4**).

**Figure 3:**
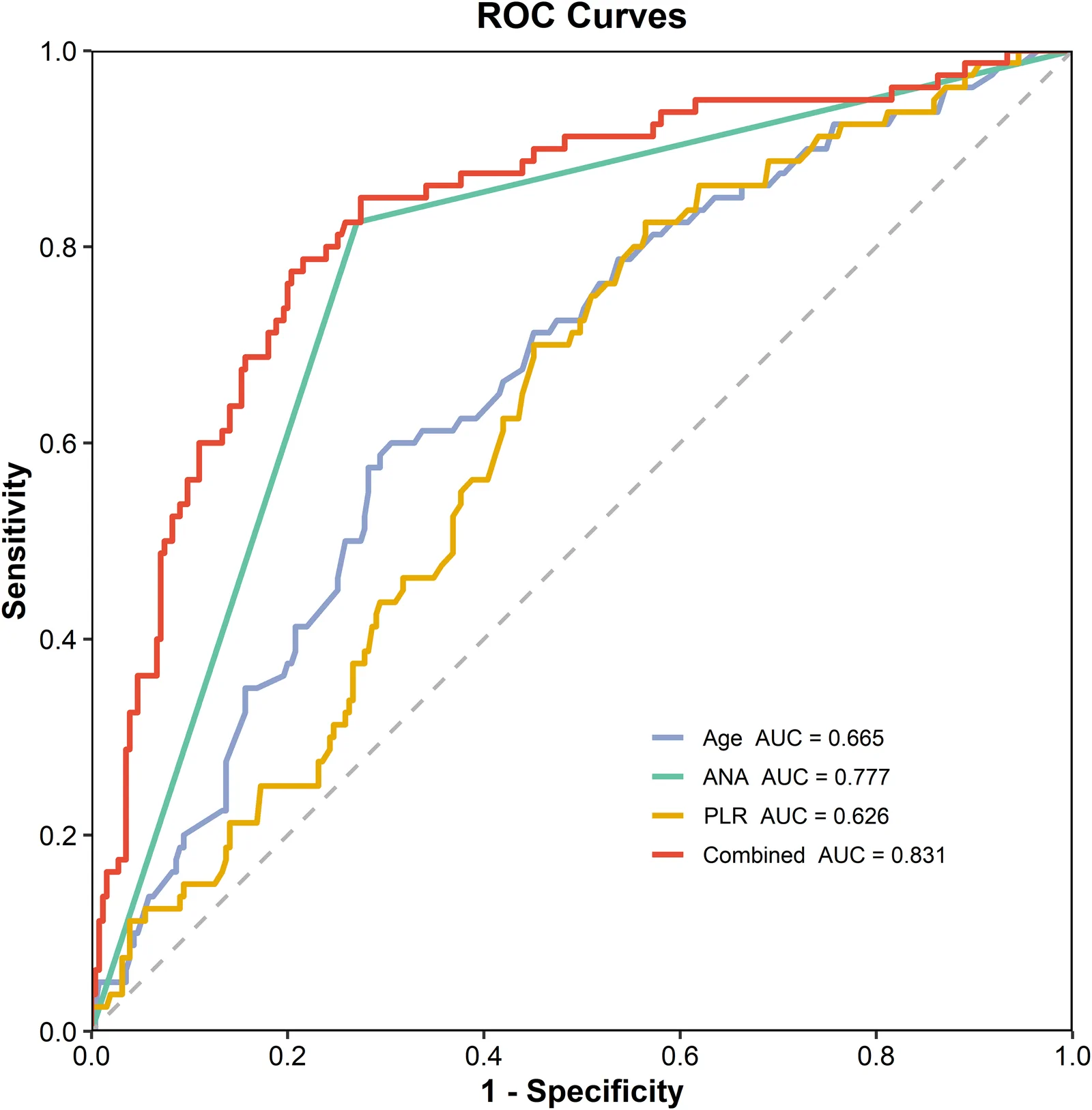
ROC curve of the combined model in the derivation cohort.

**Figure 4:**
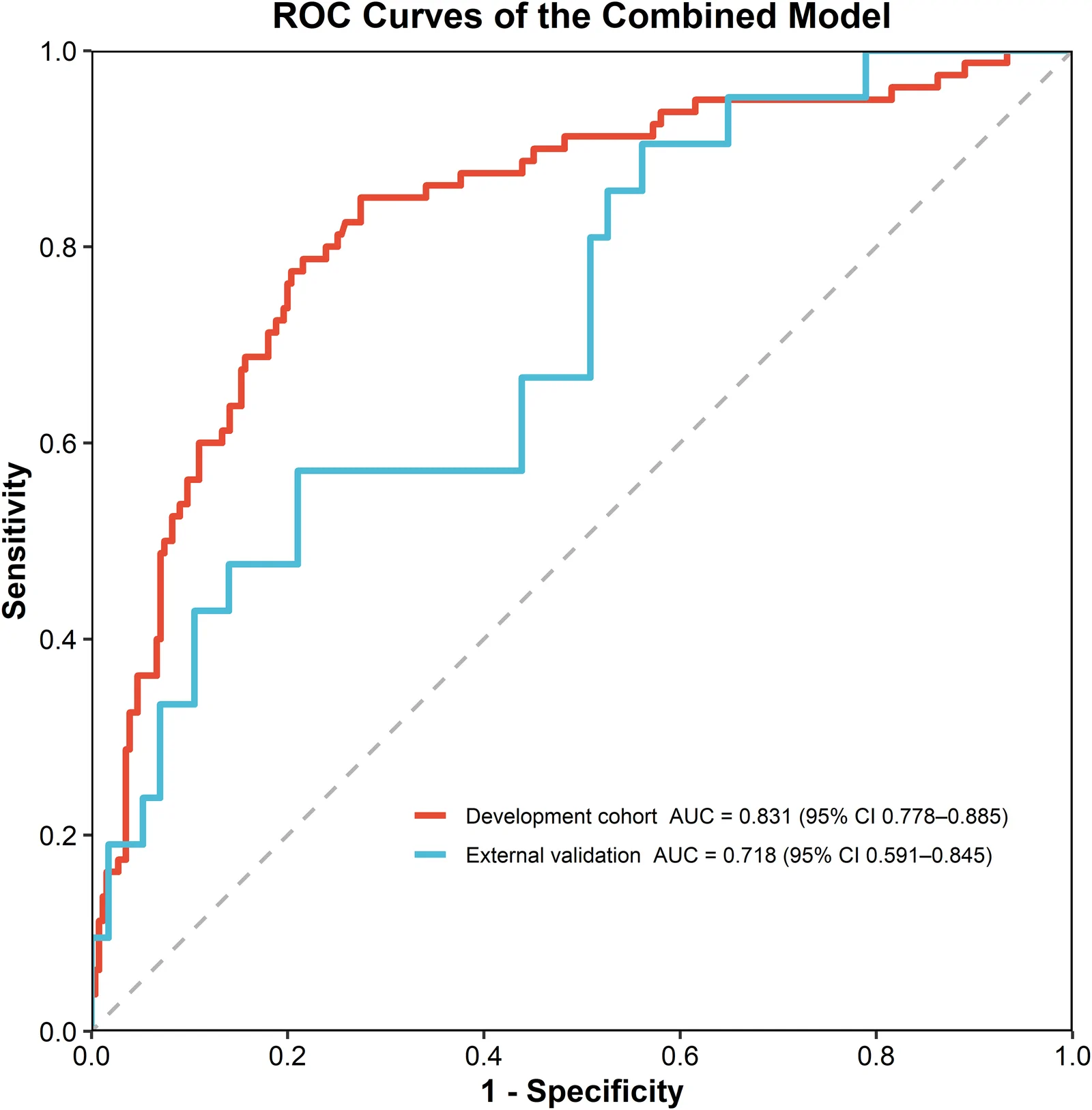
ROC curve of the combined model in the external validation cohort.

### 3.6 Calibration and decision curve analysis

Calibration curves showed generally good agreement between predicted and observed outcomes in the derivation cohort, while some local deviations were observed in the validation cohort. The Hosmer–Lemeshow goodness-of-fit test was non-significant in both the derivation cohort (χ² = 6.340, P = 0.609) and the validation cohort (χ² = 10.688, P = 0.220). The corresponding Brier scores were 0.129 and 0.137, respectively (**Figure 5**). Decision curve analysis showed that the model provided a positive net benefit across a clinically relevant threshold probability range, approximately 0.10–0.60 in the derivation cohort, with a similar trend observed in the validation cohort (**Figure 6**).

**Figure 5:**
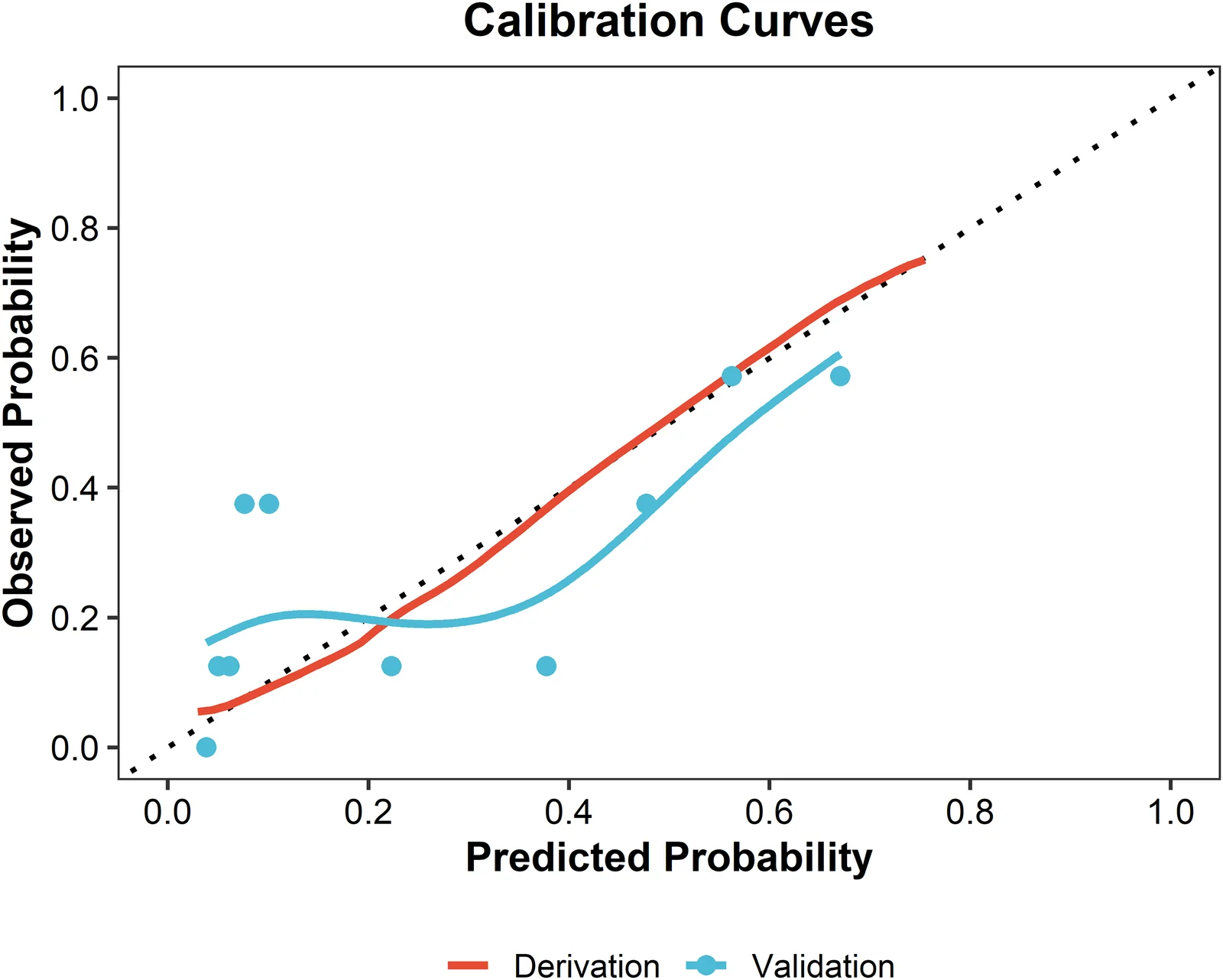
Calibration curves of the prediction model.

**Figure 6:**
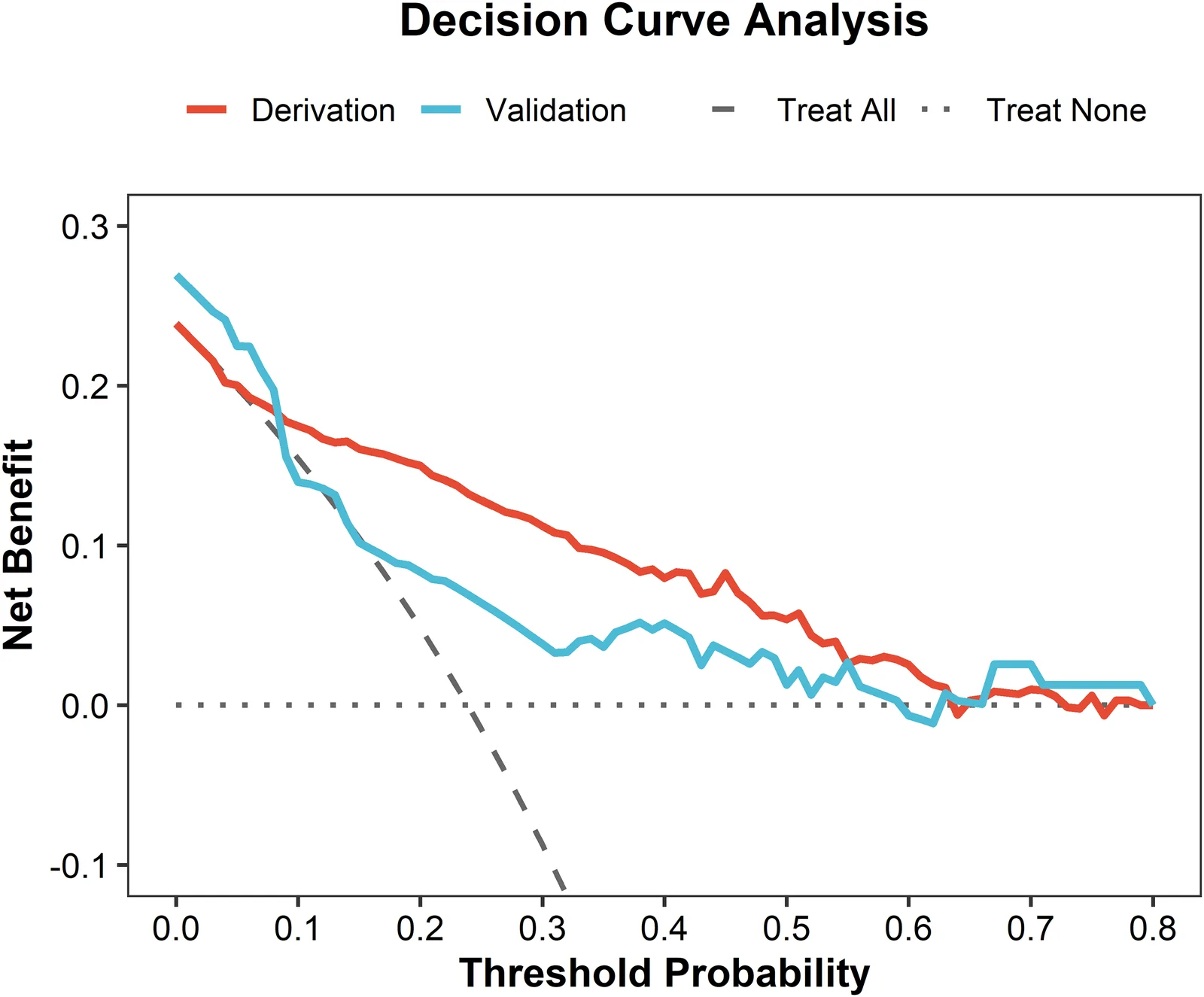
Decision curve analysis of the prediction model.

## 4 Discussion

ITP is an acquired autoimmune disorder mainly caused by increased platelet destruction and impaired platelet production (25, 26). Most children receiving early glucocorticoid therapy achieve remission; however, some patients may still exhibit an inadequate early treatment response, requiring adjustment of the therapeutic regimen and, in some cases, eventually progressing to chronic immune thrombocytopenia (27). Previous studies have shown that although severe bleeding events are relatively uncommon in children with ITP, concerns among patients and their families regarding bleeding risk and treatment-related adverse effects may lead to repeated medical visits and impose substantial psychological and financial burdens (28). Previous research has mainly focused on predictors of chronic ITP and treatment response to specific therapeutic strategies. Older age has been considered to be associated with persistent or chronic disease, while ANA/A-ENA abnormalities have been associated with poor short-term treatment response (29). In addition, studies have suggested that PLR, CD4/CD8 ratio, C3, and IgG may be associated with glucocorticoid treatment failure in children with ITP. However, evidence for predicting early treatment response in children receiving glucocorticoid therapy remains limited, and external validation is still insufficient. In this context, the present study was conducted using a regional multicenter cohort of children with ITP and comprehensively evaluated clinical, hematological, immunological, and inflammatory parameters. We found that older age, higher PLR, and ANA positivity were independently associated with early non-response to first-line treatment, and the discriminatory performance of the combined model was further evaluated in an independent validation cohort.

Our study found that an elevated PLR was independently associated with early non-response to glucocorticoid treatment in children with primary ITP (OR = 1.622), indicating that each 1-unit increase in PLR was associated with an approximately 62.2% increase in the odds of early treatment non-response. PLR is derived from the platelet count and lymphocyte count and may, to some extent, provide an integrated reflection of platelet changes and lymphocyte-mediated immune status The development and progression of ITP are closely related to complex immune dysregulation. In addition to autoantibody-mediated platelet destruction, its pathogenesis also involves activation of autoreactive T and B lymphocytes, enhanced Th1/Th17 responses, and impaired regulatory T-cell function (30, 31). Abnormalities in lymphocyte number and function may lead to impaired immune tolerance and persistent autoimmune responses, thereby affecting platelet destruction and treatment response (32). Therefore, a higher PLR may reflect an imbalanced immunohematologic state between platelets and lymphocytes, particularly relative lymphopenia and immune dysregulation, which may be associated with a poorer early treatment response. This finding is generally consistent with previous studies on blood cell-derived inflammatory indices and treatment response in ITP. Zhang et al. (33) reported that elevated PLR was independently associated with glucocorticoid treatment failure in children with primary ITP, with each 1-unit increase in PLR corresponding to an approximately 2%–13% increase in the odds of treatment failure. The same study also found that elevated IgG was associated with IVIG treatment failure (OR = 1.16), with an effect size comparable to that observed for PLR in our study. In addition, Elmeazawy et al. (34) found that elevated PLR was independently associated with chronic ITP in children (OR = 1.499), indicating that each 1-unit increase in PLR was associated with a 49.9% increase in the odds of chronic disease, further suggesting that PLR may be associated with an unfavorable disease course in pediatric ITP. However, some studies have not identified a significant association between PLR and glucocorticoid treatment response. These discrepancies may be related to differences in study populations, age distribution, treatment regimens, outcome definitions, and sample size. Therefore, the predictive value of PLR for early treatment response in pediatric ITP requires further validation.

Our study found that increasing age was independently associated with early non-response to first-line treatment in children with primary ITP (OR = 1.769), indicating that each 1-year increase in age was associated with an approximately 76.9% increase in the odds of early treatment non-response. Age may reflect differences in the immune phenotype and natural disease course of pediatric ITP. As children grow older and the immune system matures, autoimmune responses, impaired immune tolerance, and autoantibody-related immune phenotypes may become more pronounced. Consequently, older children may exhibit more persistent immune-mediated platelet destruction and a relatively poorer early treatment response. This finding is broadly consistent with previous studies. Zhang et al. (35) reported that younger children with ITP had higher complete response rates at 7 days, whereas increasing age was independently associated with poorer long-term disease outcomes (OR = 1.225), with an effect direction and magnitude similar to those observed in our study. Pregeljc et al. (15) further found that each 1-year increase in age was associated with an approximately 8% increase in the odds of progression to chronic ITP (OR = 1.08). Ren et al. reported that children aged ≥3 years had a 3.792-fold higher odds of developing chronic ITP. In addition, a systematic review and meta-analysis including 54 studies showed that age ≥11 years was significantly associated with chronic ITP (OR = 2.47). Bennett et al. (36) using data from the large international ICIS Registry II cohort, likewise found that younger age was associated with a greater likelihood of remission at 12 and 24 months; children aged <1 year had 4.7-fold and 7.0-fold higher odds of remission at 12 and 24 months, respectively. Although some of these studies focused on long-term remission or chronicity rather than early treatment response, they consistently suggest that age is an important clinical factor in the disease course of pediatric ITP. Our findings further indicate that the influence of age may already be evident during the early phase after first-line treatment, suggesting that older children with newly diagnosed ITP may require closer monitoring of early treatment response.

Our study found that ANA positivity was independently associated with early non-response to first-line treatment in children with primary ITP (OR = 3.142), indicating that ANA-positive children had approximately 3.14-fold higher odds of early treatment non-response than ANA-negative children. ANA is an important serological marker of autoimmune abnormalities, and its positivity may indicate a broader disruption of immune tolerance rather than an immune response directed solely against platelets. The pathogenesis of ITP is closely associated with autoreactive B-cell antibody production, T-cell immune dysregulation, and impaired immune tolerance (37, 38). Therefore, ANA positivity may represent an ITP immunophenotype characterized by a higher degree of autoimmune activation. This finding is broadly consistent with previous studies in pediatric ITP. Liu et al. (39) studied 1, 330 children with primary ITP and found that ANA/A-ENA abnormalities were associated with the clinical course and treatment response of the disease. In particular, children with A-ENA positivity were more likely to develop persistent or chronic ITP and showed poorer short-term treatment responses, suggesting that autoantibody abnormalities may be associated with suboptimal early treatment efficacy. Yang et al. (40) reported that persistent ANA negativity was an independent predictor of spontaneous remission (HR = 6.12), indicating that children who remained ANA-negative were substantially more likely to achieve spontaneous remission. This finding indirectly supports an association between persistent ANA positivity and poorer disease remission as well as sustained immune abnormalities. These observations are consistent with our finding that ANA-positive children had approximately 2.15-fold higher odds of early treatment non-response, suggesting that ANA may not only reflect the autoimmune status of pediatric ITP but may also have potential value in assessing early treatment response and disease course.

Overall, our findings suggest that early treatment response to first-line therapy in children with primary ITP may be influenced by multiple factors, including age, immune-inflammatory status, and autoimmune abnormalities. We found that older age, higher PLR, and ANA positivity were independently associated with early treatment non-response. All of these factors can be obtained from routine clinical and laboratory assessments before treatment initiation and therefore have good clinical accessibility. The combined model incorporating age, PLR, and ANA showed good discriminatory ability in the derivation cohort and was further evaluated in an independent external validation cohort. Accordingly, the combined assessment of these indicators may provide useful information for early treatment-response evaluation, risk stratification, and individualized clinical management in children with newly diagnosed ITP.

This study has several limitations. First, as a multicenter retrospective cohort study, patient selection and data collection relied on existing electronic medical records, which may have introduced selection bias and information bias. Therefore, the generalizability of our findings requires further validation in prospective cohorts. Second, the overall sample size was relatively small (n=335), and no *a priori* sample size calculation was performed; the sample size assessment was conducted *post-hoc* using the Riley et al. framework (23, 24). In addition, the external validation cohort included only 78 children with fewer than 20 endpoint events, which may limit the robustness of the external validation. The AUC in the external validation cohort (0.718) was lower than in the derivation cohort (0.831), which may reflect differences in case mix and treatment protocols between the two cohorts, and some degree of overfitting cannot be excluded. Direct comparison with previously published prediction models was not possible due to the lack of comparable models in pediatric ITP. Third, due to the retrospective nature of this study, we were unable to adjust for first-line treatment regimens and center-specific effects in the multivariable model. In addition, detailed dosage regimens and potential variability in treatment across patients could not be fully accounted for, which may introduce residual confounding. Future prospective studies with standardized treatment protocols are needed. Fourth, our analyses were mainly based on pretreatment baseline clinical, hematological, and immunological parameters, and we did not further evaluate dynamic changes in platelet counts, PLR, or other immune-inflammatory indicators during treatment. Given the dynamic nature of treatment response in pediatric ITP, serial monitoring of relevant indicators may help further improve the accuracy of early response assessment. In addition, the predictors included in this study were mainly derived from routine clinical and laboratory tests, and more detailed immunological parameters and cytokine profiles were not assessed, although these factors may be involved in immune dysregulation and treatment response in ITP. Meanwhile, predictor selection in this study partly relied on data-driven methods, including correlation analysis and LASSO regression; therefore, the selected predictors and their regression coefficients may still be sample-dependent. In addition, we did not compare logistic regression with machine learning algorithms (e.g., Random Forests, Support Vector Machines, or Gradient Boosted Decision Trees), as complex models are prone to overfitting in small samples. This may be considered a limitation. Fifth, due to the limited sample size, subgroup analyses stratified by treatment regimen, age category, or ethnicity were not performed; this may be considered a limitation. Sixth, all samples were derived from two centers in the Xinjiang region, encompassing a multi-ethnic population. Ethnic differences in immune characteristics may influence the predictive effects of immunological parameters, and caution should be exercised when extrapolating our findings to other populations. A nomogram or risk scoring system was not developed; we instead provided the full model specification to allow manual calculation of the risk score. Finally, future studies with larger, multicenter, and prospective cohorts are needed to further validate the stability of the model and to incorporate dynamic hematological and immunological indicators to improve the prediction of early treatment response in pediatric ITP.

## Data Availability

The data analyzed in this study is subject to the following licenses/restrictions: The datasets are not publicly available due to patient privacy and ethical restrictions. De-identified data may be made available from the corresponding author upon reasonable request and subject to approval by the relevant institutional and ethics committees. Requests to access these datasets should be directed to yan10mei25@163.com.

## Supplementary material

Supplementary Table 1 Detailed comparison of baseline characteristics between the early treatment responder and non-responder groups.

Supplementary Table 2 Multivariable logistic regression analysis of predictors selected by LASSO for early treatment non-response.
