# Supplementary material for: Risk factors analysis and validation of early treatment non-response in children with primary ITP: a multicenter retrospective cohort study
Source: Front Immunol. 2026 Oct 7;17:1983623. doi: 10.3389/fimmu.2026.1983623 (PMC13683604; doi:10.3389/fimmu.2026.1983623)
Supplement: Supplementary file 1 [file Table1.docx]

**Supplementary Table S1. Detailed comparison of baseline characteristics between the early treatment responder and non-responder groups**

| Characteristic | Overall  N = 335 | R group  N = 255 | NR group  N = 80 | p-value |
| --- | --- | --- | --- | --- |
| Ethnicity |  |  |  | 0.111 |
| Uyghur | 201 (60.0%) | 160 (62.7%) | 41 (51.3%) |  |
| Han | 88 (26.3%) | 60 (23.5%) | 28 (35.0%) |  |
| Other | 46 (13.7%) | 35 (13.7%) | 11 (13.8%) |  |
| Season |  |  |  | 0.098 |
| Spring | 96 (28.7%) | 65 (25.5%) | 31 (38.8%) |  |
| Summer | 124 (37.0%) | 96 (37.6%) | 28 (35.0%) |  |
| Autumn | 35 (10.4%) | 30 (11.8%) | 5 (6.3%) |  |
| Winter | 80 (23.9%) | 64 (25.1%) | 16 (20.0%) |  |
| Vaccination |  |  |  | 0.204 |
| No | 328 (97.9%) | 248 (97.3%) | 80 (100.0%) |  |
| Yes | 7 (2.1%) | 7 (2.7%) | 0 (0.0%) |  |
| Familial history of ITP |  |  |  | >0.999 |
| No | 328 (97.9%) | 249 (97.6%) | 79 (98.8%) |  |
| Yes | 7 (2.1%) | 6 (2.4%) | 1 (1.3%) |  |
| Splenomegaly |  |  |  | >0.999 |
| No | 331 (98.8%) | 252 (98.8%) | 79 (98.8%) |  |
| Yes | 4 (1.2%) | 3 (1.2%) | 1 (1.3%) |  |
| Lymphadenopathy |  |  |  | 0.866 |
| No | 287 (85.7%) | 218 (85.5%) | 69 (86.3%) |  |
| Yes | 48 (14.3%) | 37 (14.5%) | 11 (13.8%) |  |
| folic_acid | 31.60 (15.78, 53.69) | 33.27 (16.92, 57.35) | 19.48 (10.90, 45.27) | <0.001 |
| Vit_B12 | 35.82 (16.52, 338.64) | 32.10 (15.93, 332.24) | 230.49 (19.80, 340.69) | 0.083 |
| Fe2 | 124.58 (42.65, 388.76) | 175.99 (47.01, 407.49) | 74.29 (38.21, 327.07) | 0.018 |
| EPO | 12.53 (8.22, 25.07) | 13.15 (8.23, 26.52) | 11.94 (7.61, 17.87) | 0.142 |
| IgM | 0.98 (0.69, 1.24) | 0.95 (0.69, 1.23) | 1.03 (0.71, 1.25) | 0.346 |
| IgE | 43.00 (17.00, 100.00) | 42.00 (17.00, 101.00) | 44.50 (18.50, 91.50) | 0.925 |
| Anti SSA |  |  |  | 0.263 |
| 0 | 178 (53.1%) | 141 (55.3%) | 37 (46.3%) |  |
| 1 | 76 (22.7%) | 53 (20.8%) | 23 (28.8%) |  |
| 2 | 81 (24.2%) | 61 (23.9%) | 20 (25.0%) |  |
| Anti Ro52 |  |  |  | 0.425 |
| 0 | 157 (46.9%) | 124 (48.6%) | 33 (41.3%) |  |
| 1 | 73 (21.8%) | 52 (20.4%) | 21 (26.3%) |  |
| 2 | 105 (31.3%) | 79 (31.0%) | 26 (32.5%) |  |
| Anti SSB |  |  |  | 0.272 |
| 0 | 330 (98.5%) | 252 (98.8%) | 78 (97.5%) |  |
| 1 | 4 (1.2%) | 3 (1.2%) | 1 (1.3%) |  |
| 2 | 1 (0.3%) | 0 (0.0%) | 1 (1.3%) |  |
| ACA |  |  |  | 0.201 |
| 0 | 322 (96.1%) | 247 (96.9%) | 75 (93.8%) |  |
| 1 | 13 (3.9%) | 8 (3.1%) | 5 (6.3%) |  |

**Supplementary Table S2. Multivariable Logistic Regression Analysis of Predictors Selected by LASSO for Early Treatment Non-response**

| variable | β | OR | 95%CI | P |
| --- | --- | --- | --- | --- |
| Age | 0.153 | 1.165 | 1.082-1.257 | <0.001 |
| PDW | -0.049 | 0.952 | 0.912-1.002 | 0.121 |
| PLR | 0.142 | 1.153 | 1.045-1.406 | 0.045 |
| ANA | 0.765 | 2.148 | 1.211-3.941 | 0.011 |
